# Drug resistance gene polymorphisms of *Plasmodium falciparum* clinical isolates from Kinshasa, Democratic Republic of the Congo

**DOI:** 10.1186/s41182-026-01022-5

**Published:** 2026-07-07

**Authors:** Mimie Bitshi, Taeko K. Naruse, Tomoyo Sakata-Kato, Héritier Nzuau Mabi, Prince Kassanda, Gloria Kangite Yakenvu, Evariste Tshibangu-Kabamba, Papy Mandoko Nkoli, Natsuko Kaku, Yu Nakagama, Yasutoshi Kido, Dieudonné Mumba Ngoyi, Osamu Kaneko

**Affiliations:** 1 Program for Nurturing Global Leaders in Tropical and Emerging Infectious Diseases, Graduate School of Biomedical Sciences, Nagasaki University, Nagasaki, Japan; 2 Department of Protozoology, Institute of Tropical Medicine (NEKKEN), Nagasaki University, Nagasaki, Japan; 3 Department of Parasitology, National Institute of Biomedical Research, Kinshasa, Democratic Republic of the Congo; 4 Department of Tropical Medicine, University of Kinshasa, Kinshasa, Democratic Republic of the Congo; 5 Department of Virology & Parasitology, Graduate School of Medicine, Osaka Metropolitan University, Osaka, Japan; 6 Research Center for Infectious Disease Sciences, Graduate School of Medicine, Osaka Metropolitan University, Osaka, Japan

**Keywords:** Drug resistance, Molecular surveillance, *Plasmodium falciparum*, Artemisinin, Kinshasa

## Abstract

**Background:**

Sustained efficacy of artemisinin (ART)-based combination therapies is essential for controlling *Plasmodium falciparum* malaria in Africa. ART derivatives remain the key frontline treatment, while sulfadoxine-pyrimethamine is used for prevention. Recent detection in Africa of *P. falciparum kelch 13* (*k13*) substitutions associated with ART resistance (ART-R), distinct from Southeast Asian *k13* ART-R alleles, suggests local emergence. Together with widespread pyrimethamine resistance, ART-R poses a major public health concern. In the Democratic Republic of the Congo (DRC), however, data on resistance markers beyond conventional loci remain limited.

**Methods:**

From June 2022 to February 2023, 303 malaria rapid diagnostic test-positive patients with uncomplicated malaria were enrolled at three clinics in Kinshasa, the Democratic Republic of the Congo (DRC). Day 1 parasitemia reduction rate (PRR) after artemether-lumefantrine treatment was assessed by microscopy. The gene region encoding the K13 propeller domain was sequenced from 171 microscopy-confirmed *P. falciparum* cases. Whole-genome sequencing data from 31 culture-adapted isolates were used to examine polymorphisms and copy number in 13 resistance-associated genes: *k13*, *coronin, kic4, kic5, kic7, px1*, *ap2-mu*, *ubp1*, *mdr1*, *mdr2*, *crt*, *dhfr*, and *dhps.*

**Results:**

No validated or candidate K13 propeller mutation associated with ART partial resistance was detected, including in samples from patients with < 80% PRR. One isolate carried K13 A578S, a variant previously shown not to confer ART resistance. Whole-genome sequencing showed that all 13 genes were single copy and revealed diverse known and novel polymorphisms. K13 Asn-repeat variants and non-*k13* candidate variants, including PX1 M1701I/D1705N, AP2-Mu S160N, and UBP1 E1528D, were detected. Partner-drug and antifolate markers included MDR1 Y184F, the CRT chloroquine-resistant IET haplotype, the DHFR N51I/C59R/S108N triple-mutant haplotype, and DHPS K540E. Among variants compared between PRR groups, the synonymous *ap2-mu* a300c substitution remained significant after false discovery rate correction.

**Conclusions:**

Established K13 propeller-mediated ART partial resistance was not evident in Kinshasa at the time of sampling. However, diverse non-*k13* candidate variants and persistent partner-drug and antifolate resistance markers support expanded molecular surveillance combined with clinical and ex vivo phenotypic monitoring.

**Supplementary Information:**

The online version contains supplementary material available at 10.1186/s41182-026-01022-5.

## Background

Malaria is a mosquito-borne tropical infectious disease caused by protozoa of the genus *Plasmodium*. Half of the world population still lives under the threat of malaria, which continues to infect hundreds of millions of people every year, with the heaviest burden noted in Africa [1]. Among *Plasmodium* species, *Plasmodium falciparum* is the deadliest species with estimated 2–3 million deaths every year. Reducing the burden of *falciparum* malaria in Africa currently relies mainly on the effectiveness of artemisinin (ART)-derivatives, which constitute the backbone of frontline treatment for both uncomplicated and severe malaria cases. However, one major challenge for malaria control and elimination efforts is ART-resistance (ART-R), which has emerged and spread over the past two decades from its origin in the Greater Mekong subregion of Southeast Asia. Recently, alarming molecular signals of a nascent ART-R have been detected in several African regions, a continent initially thought to be spared [2]. The emergence of ART-R in African parasites constitutes a troubling scenario that can have severe consequences on the global burden of malaria because alternative therapies are few. Tracking the emergence of resistant parasites in Africa is thus crucial from a global health perspective. Substitutions in *kelch 13* (*k13*) gene identified as the molecular markers of ART-R in Southeast Asia in 2014 provided useful insights for surveillance in Africa as well [3]. However, the biological relevance of these markers and that of several African-specific substitutions noted in this gene still requires local validation in African settings. Furthermore, clinical ART-R has been reported in Africa in the absence of known K13 ART-R substitutions, suggesting the presence of K13-independent ART-R mechanisms that might differ from those of Southeast Asian parasites [2, 4]. For instance, the *P. falciparum coronin* gene, phosphoinositide-binding protein gene (*px1*), adaptor protein complex 2 mu subunit gene (*ap2-mu*), and ubiquitin-binding protein 1 gene (*ubp1*) have been shown to be linked to ART-R, suggesting that non-*k13* type of resistance may independently emerge in natural settings in Africa [5–10].

The Democratic Republic of the Congo (DRC) ranks second among the most affected by malaria worldwide, with over 35 million cases and 67 thousand deaths recorded in 2024 [1]. In DRC, almost all malaria cases are caused by *P. falciparum*, which displays a stable transmission over the entire year and threatens the entire population [11]. The emergence and spread of antimalarial drug resistance remain a significant concern, continually threatening the progress made in malaria control over the last decade. Intermittent preventive treatment with sulfadoxine-pyrimethamine (SP) is recommended since 2006 for pregnant women [11]. However, resistance to SP as well as chloroquine (CQ) has been reported at very high levels in the country for several years [12]. The development of resistance to ART-derivatives is a matter of grave concern also in the DRC. First, Ashley et al. (2014) reported that one among 59 *P. falciparum* malaria patients (2%) in Kinshasa still showed parasitemia on day 3 after treatment with artesunate, suggesting clinical ART-R, although known ART-R K13 substitutions were not detected [13]. Then, parasites carrying *k13* with novel ART-R substitutions have been reported in neighboring countries, such as Rwanda (R516H) and Uganda (A675V and C469Y) [2, 14, 15], and later in bordering regions of the DRC [16, 17]. However, for monitoring ART-R, the National Malaria Control Program in the DRC continues to rely primarily on therapeutic efficacy trials, which, although considered the standard approach, are constrained by the high cost of implementation and the potential for outcome bias arising from population-level acquired immunity to malaria [18, 19]. Therefore, a more efficient system for epidemiological surveillance of ART-R should integrate routine monitoring of molecular markers; however, this is still lacking in the country, and little is known about the molecular profile of the parasite population. The DRC's central location in Africa, combined with its substantial malaria burden, suggests that improving our understanding of malaria epidemiology in the country is essential for effective malaria control across the continent. Previous studies in the DRC have predominantly examined resistance-associated variants in *crt*, *dhfr*, *dhps*, and *k13.* In this study, in addition to these molecules, we aimed to evaluate polymorphisms of the other ART-resistance-associated genes in *P. falciparum* isolated from malaria patients in a high-transmission area in Kinshasa, using a WGS strategy, addressing a critical knowledge gap in surveillance of malaria molecular markers [20]. This study will provide critical information for drug policymakers and enable the development of more effective control interventions.

## Methods

### Ethical statement

This study was approved by the National Ethics Committee of the Ministry of Public Health, Hygiene, and Prevention of the Democratic Republic of the Congo, as well as the Research Ethics Committee of the Institute of Tropical Medicine, Nagasaki University, Japan. Written informed consent was obtained from adult patients who voluntarily agreed to participate in this study, while parents or guardians provided consent for their children.

### Study site

**Fig. 1:**
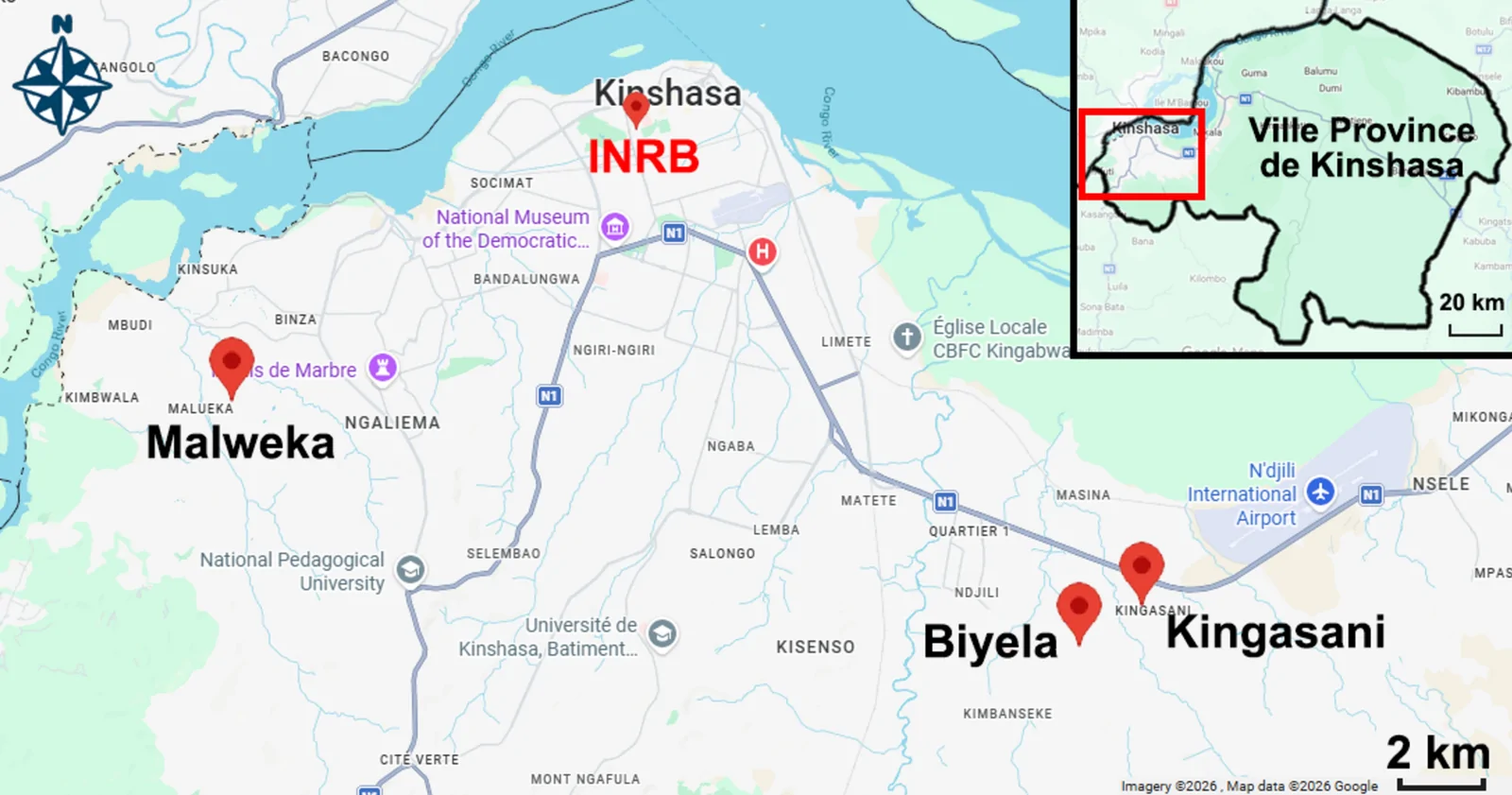
Locations of the three hospitals in Kinshasa where patient samples were collected. Locations of the hospitals at Kingasani, Biyela, and Malweka, and of the National Institute of Biomedical Research (INRB), where the samples were stored are shown. In the inset, the red box on the map of Kinshasa Province indicates the area enlarged in the main panel

The blood samples were obtained from patients who visited three clinics, Kingasani Hospital Center, Saint Joseph Biyela Hospital Center, and the Reference Hospital of Malweka in Kinshasa, the Democratic Republic of Congo, from June 2022 to February 2023 (Fig. 1). These sites have a high malaria prevalence and are located in three different Health Zones, with Kingasani Hospital Center serving as a sentinel site for malaria surveillance in Kinshasa.

### Blood collection

Malaria was diagnosed by rapid diagnostic tests (RDT, Bioline Malaria Ag P.f test, Abbott Diagnostics Korea Inc., Seoul, Korea). Pregnant individuals and those who had taken antimalarial drugs within the previous four weeks were excluded. RDT-positive patients seeking care due to malaria-related symptoms without signs of severe disease (uncomplicated malaria) were invited to donate 4 mL of venous blood using EDTA vacutainers before receiving artemether-lumefantrine (AL) on the day of admission (day 0). Of this volume, 3 mL was aseptically mixed with 5 mL of RPMI-1640 medium containing 0.25% AlbuMAX II, 5% heat-inactivated pooled AB^+^ human fibrinogen-free human plasma, 200 μM hypoxanthine (Sigma-Aldrich, St. Louis, MO, US), and 10 µg/mL gentamicin (Sigma-Aldrich) (complete medium) and placed with ice packs until cryopreservation. The remaining 1 mL of blood was used to prepare Giemsa-stained smears and dried blood spots (DBS) on FTA filter paper (QIAcard FTA Classic, Qiagen, Hilden, Germany). One day after treatment (day 1), additional DBS and Giemsa-stained smears were obtained from each participant, and day 1 parasitemia was evaluated by microscopy.

The parasitemia reduction rate (PRR) was determined by calculating the ratio of day 1 to day 0 parasitemia. Isolates were divided into two groups; < 80% PRR and ≥ 80% PRR. Because early parasite clearance after AL treatment is likely influenced more by rapidly acting artemether than by slowly absorbed lumefantrine, we assessed day 1 parasitemia reduction as a possible indicator of artemisinin resistance. It has been shown that more than 90% of parasites are typically cleared within 24 h following artesunate monotherapy [21]. The ART-R–type K13 substitution was observed only in samples with low day 1 reduction in the study conducted in Kenya, suggesting that this measure may be useful for identifying candidate artemisinin-resistant parasites [22].

### *P. falciparum* culture

The blood was thawed in the laboratory and incubated at 2% haematocrit with O^+^ red blood cells (RBCs) in complete medium at 37 °C under a gas mixture of 5% CO_2_, 5% O_2_, and 90% N_2_ to adapt infected *P. falciparum* to in vitro culture conditions. Human RBCs and plasma were obtained from Japanese Red Cross Society with approval from the Research Ethics Committee, Institute of Tropical Medicine, Nagasaki University.

### Semi-nested PCR and sequencing of *k13* gene locus

Genomic DNA (gDNA) was extracted from DBS using the QIAmp DNA Mini Kit (Qiagen). The DNA fragments encoding the propeller domain of K13 (nucleotide positions 440–726) were amplified. First round PCR was performed in a 50 μL reaction mixture with primers *PfK13*.F3 (AGTGGAAGACATCATGTAACCAG) and *PfK13*.R3 (TGTGCATGAAAATAAATATTAAAGAAG) using TKS Gflex DNA polymerase (Takara Bio, Shiga, Japan) to amplify a 1143-bp fragment. Cycling conditions were as follows: initial denaturation at 98 °C for 1 min; 29 cycles of 98 °C for 10 s, 60 °C for 15 s, and 68 °C for 30 s, and a final elongation at 72 °C for 5 min. The second-round PCR used primers *PfK13*.F3 and *PfK13*.R2 (CGGAGTGACCAAATCTGGG), with 0.5 μL of the first-round PCR product as a template solution, under the same conditions to amplify a 1021-bp fragment. The nested PCR products were purified using ExoSAP-IT™ Express PCR Product Cleanup (Thermo Fisher Scientific, Waltham, MA, US) and sequenced using *PfK13*.F1 (GCCTTGTTGAAAGAAGCAGAA) and *PfK13*.R1 (GCCAAGCTGCCATTCATTTG). Sequences were aligned by SnapGene software (ver 7.2.1; GSL Biotech LLC, Boston, MA, US) using the PF3D7_1343700 sequence as a reference.

### Whole genome sequencing (WGS)

Cultured RBC samples, including parasite-infected RBCs, were harvested from cultures at least 20 mL maintained at 2% hematocrit with > 5% parasitemia. The samples were incubated with 0.15% saponin in PBS on ice for 5 min and washed with PBS, and gDNA was subsequently extracted from the parasite pellets using QIAmp DNA Mini Kit (Qiagen). WGS was performed using 2 μg of gDNA on an Illumina NovaSeq 6000 (Illumina, San Diego, CA, US) and deposited in the DDBJ under the BioProject number PRJDB39668. Sequences were trimmed from each acquired WGS FASTQ dataset and aligned to the 3D7_1343700 genome dataset as the reference. The nucleotide sequences corresponding to the 13 target genes extracted from the WGS data set of 31 samples were aligned and amino acid sequences were predicted using the Genetyx^®^ ver.17 (GENETYX Corporation, Japan). In this manuscript, nucleotide substitutions are displayed with lowercase letters as “nucleotide of 3D7 strain + nucleotide position in the open reading frame + nucleotide of the target strain.” and amino acid substitutions are displayed with uppercase letters as “amino acid of 3D7 strain + amino acid position + amino acid of the target strain”. Copy number variations (CNVs) were evaluated by comparing read depth. For each of the 13 genes detected in the WGS data from 31 samples, the mean read depth was calculated across three conserved consecutive 100-bp regions. The resulting values were normalized to the overall genome-wide coverage and compared with those of the corresponding regions in the reference genome of the *P. falciparum* 3D7 strain. To minimize the influence of sequencing and mapping errors, only nucleotides supported by ≥ 10% of reads covering each polymorphic site were included in the allele frequency analysis.

### Statistical analysis and software

Data analysis was performed using Excel (Microsoft, Redmond, WA, US) and Prism 10 (GraphPad Software, Boston, MA, US). Chi-square test or two-tailed Fisher's exact test was used to compare clinical and demographic characteristics among the study population. Spearman’s rank test was used to assess the correlation between age and body temperature. To assess the difference in allele frequencies between the ≥ 80% and < 80% PRR groups, two-tailed Fisher's exact test was used. For indels, variants of the same type relative to 3D7 were pooled: all insertions were combined when only insertions were observed, and all deletions were combined when only deletions were observed, regardless of the number or identity of the inserted or deleted amino acids. The frequencies of indel-carrying and 3D7-type clones were then compared. When both insertion- and deletion-type indels were observed relative to 3D7, clones carrying insertions or deletions were pooled separately, regardless of the number or identity of the inserted or deleted amino acids. The frequency of each indel type was then compared with that of 3D7-type clones. *P* value less than 0.05 was considered significant. Because *dhfr-ts* and *pppk-dhps* genes were not investigated in relation to artemisinin resistance, multiple testing correction was performed using the Benjamini–Hochberg false discovery rate method based on the polymorphic sites identified in the remaining 11 genes. Linkage disequilibrium was assessed using r^2^, calculated from the 2 × 2 allelic contingency table using Microsoft Excel.

## Results

### Blood sample collection, microscopy assessment, and participants' characteristics

**Fig. 2:**
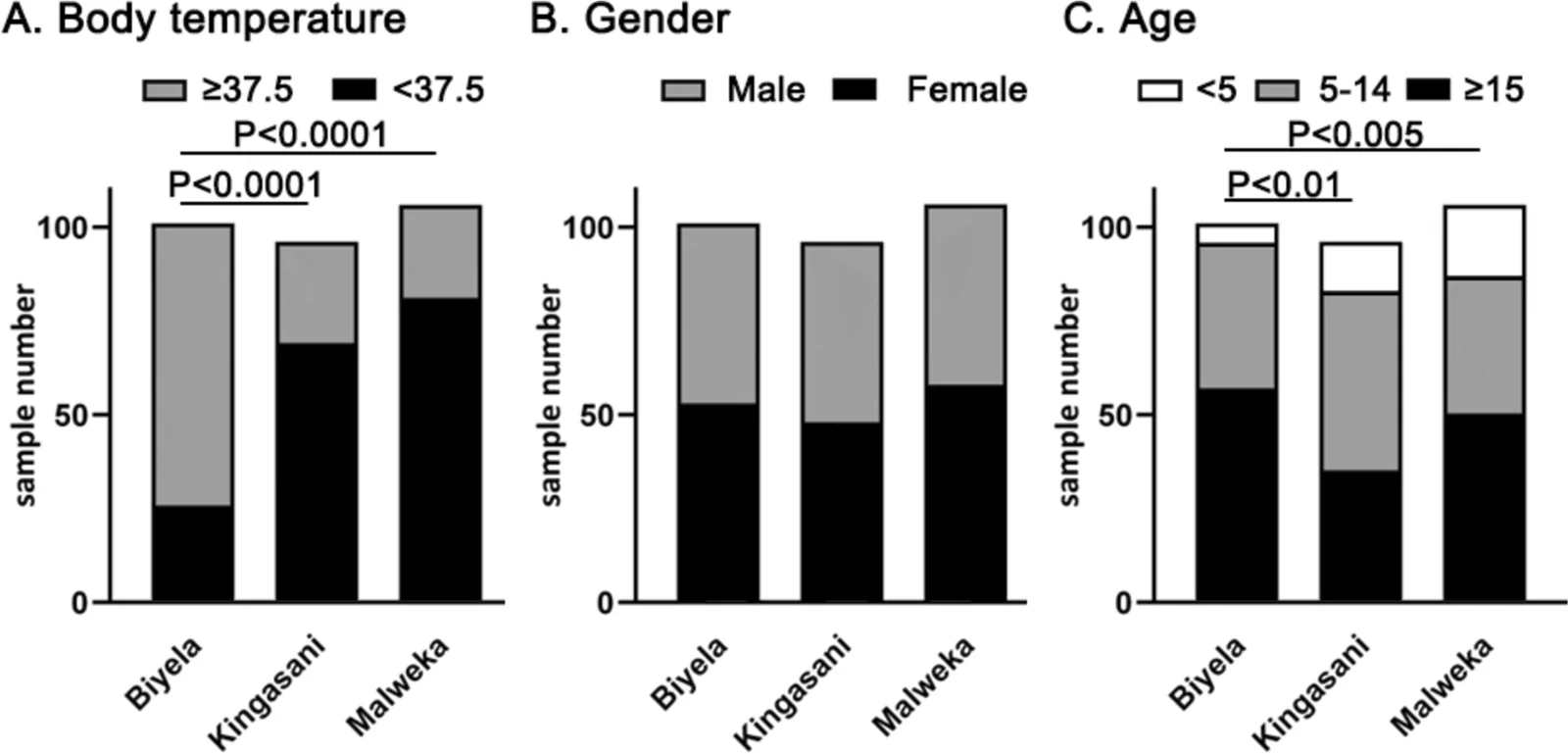
Sociodemographic and clinical characteristics. Patients at each site were stratified by **A** body temperature (≥ 37.5 °C vs < 37.5 °C), **B** gender, and **C** age group (< 5 years, 5 to 14 years, and ≥ 15 years). *P* values are shown when a significant difference in proportions was detected using the chi-square test or Fisher’s exact test

Between June 2022 and February 2023, a total of 303 malaria RDT-positive blood samples were collected from patients attending three clinics in Kinshasa, DRC. Saint Joseph Biyela Hospital Center had a significantly higher proportion of patients presenting with body temperature ≥ 37.5 °C (78.8%) compared to Kingasani Hospital Center (28%) and the Reference Hospital of Malweka (21%) (*P* < 0.0001, Fig. 2A). No significant differences in parasite carriage were observed between males and females across the three sites (Fig. 2B). When the patients were classified into three age groups (< 4 years, 5–14 years, and ≥ 15 years), the age-group distribution differed significantly between Biyela and Kingasani (*P* < 0.01) and between Biyela and Malweka (*P* < 0.005) (Fig. 2C). However, no significant correlation was detected between age and body temperature using Spearman’s rank correlation. Thus, the observed difference in fever frequency between sites may reflect site-specific factors, such as the potential high malaria transmission intensity in Biyela, the presence of other illnesses, and so on. Microscopic examination detected parasites from 189 samples (62.4%) among 303 RDT-positive samples. Among 189 samples, *P. falciparum* was detected in 183 (96.8%), including 6 mixed infection with *P. malariae*. *P. malariae* was detected from 10 samples (5.3%), including the above-mentioned mixed infection with *P. falciparum*. Two samples were *P. ovale* (1.1%) positive. The remaining 114 samples were negative by microscopy despite being RDT-positive, which may reflect submicroscopic infections, persistent antigen detection following treatment, or variability in sample quality. Among 177 patients who were positive for *P. falciparum*, 11 (6.2%) did not achieve an 80% reduction in parasitemia one day after treatment with artemether-lumefantrine (AL). These patients were categorized as the < 80% PRR group, representing delayed early parasite clearance; this grouping was used for exploratory genetic association analysis and was not regarded as confirmed clinical artemisinin resistance. In the remaining 166 cases, parasitemia was reduced by 80% or more one day after treatment with AL (≥ 80% PRR group). The distribution of *Plasmodium* species by site and the outcomes of parasitemia reduction are summarized in Tables 1 and 2.

**Table 1:** *Plasmodium* species identification by microscopy

Site	Total	*Pf*	*Pm*	*Po*	*Pf* + *Pm*	Microscopy (–)
Biyela	101	63	1	1	4	32
Kingasani	96	57	1	0	2	36
Malweka	106	57	2	1	0	46
Total	303	177	4	2	6	114

*Pf*, *Pm*, and *Po* indicate *P. falciparum*, *P. malariae*, and *P. ovale*, respectively

**Table 2:** Day 1 parasitemia reduction rate (PRR) by site

Site	Total	< 80% PRR	≥ 80% PRR
Biyela	63	6	57
Kingasani	57	1	56
Malweka	57	4	53
Total	177	11	166

### Reported K13 ART-R substitutions were not found from 171 DBS samples

To investigate the presence of known ART-R type K13 substitutions, the DNA fragment encoding K13 propeller domain was amplified using gDNA extracted from 177 DBSs obtained from patients microscopically positive for *P. falciparum* (11 DBSs on day 1 for samples showing < 80% PRR and 166 DBSs on day 0 for samples showing ≥ 80% PRR) and sequenced. Because we assumed that, in mixed infections with resistant and sensitive clones, resistant clones would be enriched and more readily detected after treatment initiation, DBS samples collected on day 1 were analyzed for samples showing < 80% PRR. Readable sequences were obtained for 171 of 177 samples (96.6%) and no validated or candidate ART-R marker nucleotide variants were detected in all samples, including 11 samples with < 80% PRR. Six samples harbored synonymous variants (c1407t, t1413c, c2070a, t2076a), with t2076a being newly identified in this study. This is the first time to detect c2070a variant in the DRC, although it had been described in Niger in 2024 [23]. Additionally, one sample from Malweka (MAL040) carried the nonsynonymous g1734t (A578S) variant, which has been reported in African and Asian countries and has been shown not to confer ART-R in transgenic parasites [24]. The summary of all identified variants is shown in Table 3. These findings indicate the continued absence of ART-R–associated substitutions within the K13 propeller domain in the study population.

**Table 3:** Substitutions found in *k13* gene region encoding the propeller domain among 171 isolates

Nucleotide change	Substitution type	Amino acid change	Frequency	Previous report [reference]
g1734t	Nonsynonymous	A578S	0.6% (1/171)	Yes [24, 25]
c1407t	Synonymous	(–)	1.2% (2/171)	Yes [44]
t1413c	Synonymous	(–)	1.2% (2/171)	Yes [44]
c2070a	Synonymous	(–)	0.6% (1/171)	Yes [23]
t2076a	Synonymous	(–)	0.6% (1/171)	No

### WGS analysis for 31 culture-adapted *P. falciparum* Kinshasa isolates

For the WGS sample size, we referred to previous small-scale studies that investigated antimalarial resistance genes and typically analyzed approximately 20 to 50 cases [13, 26]. In total, 9 of the 11 *P. falciparum* isolates from the < 80% PRR group and 22 isolates from the ≥ 80% PRR group were culture-adapted to enable downstream genomic analyses from a large amount of gDNA. Building on the initial analysis of the K13 propeller domain by Sanger sequencing, WGS was performed on these 31 culture-adapted Kinshasa isolates. The average read depth ranged between 100 and 250 reads, and the average Q30 of 90.6% (88.7–95.3%) indicated a high quality of the obtained sequence data set (Figure S1).

Using the copy number of each gene in the 3D7 genome as a reference value of 1, all 13 genes analyzed in this study in all 31 samples showed normalized values between 0.5 and 1.5, suggesting that each gene was present as a single copy in all samples. In all isolates, SNPs with a minor allele frequency of ≥ 10% were identified in at least one gene among all 13 genes analyzed in this study. Accordingly, each isolate was considered to represent a mixed parasite population and 31 isolates were conservatively treated as comprising 62 clones.

### Substitutions of eight genes linked with ART-R

**Fig. 3:**
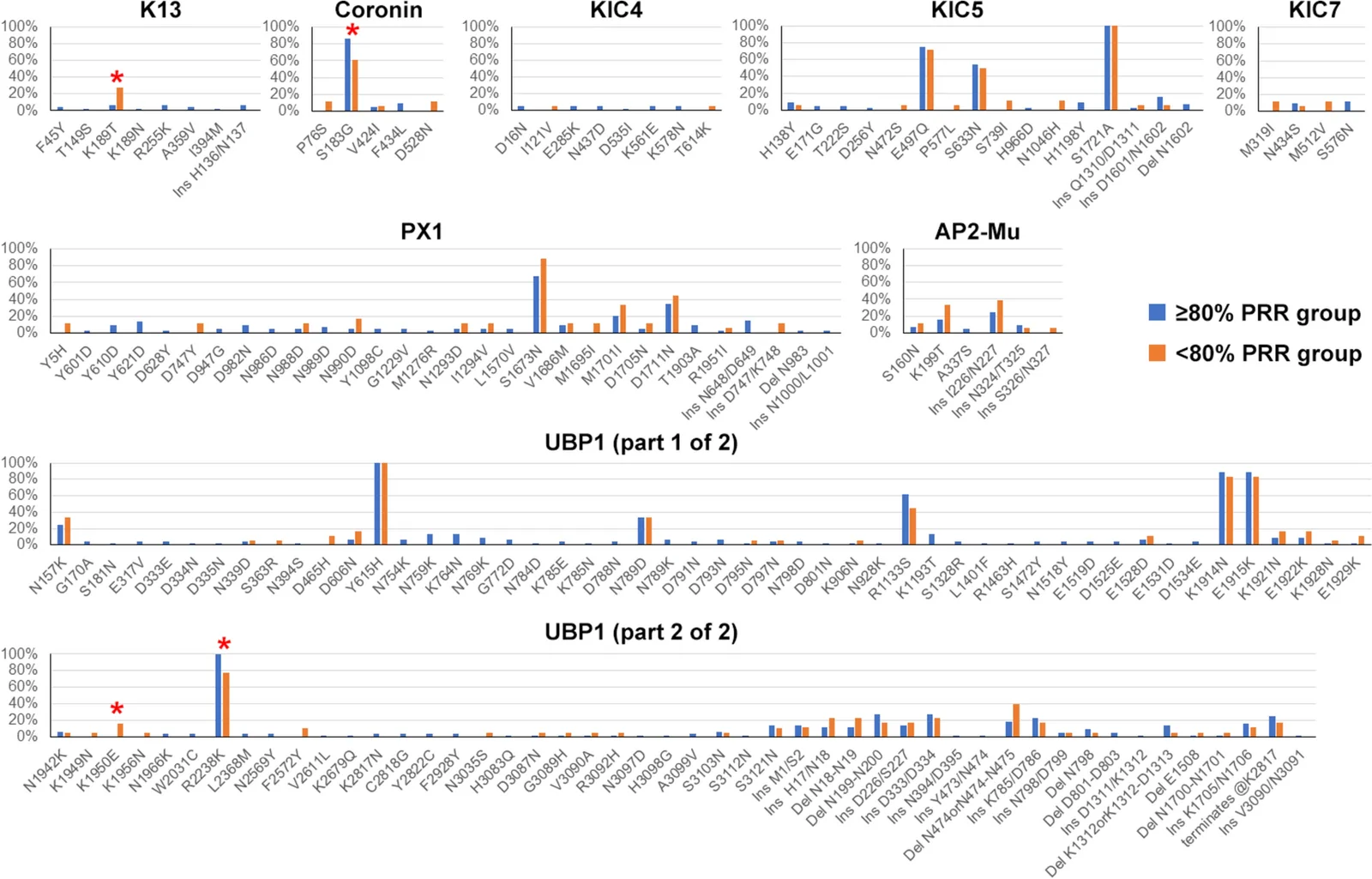
Frequencies of parasite clones carrying amino acid substitutions or indels in eight putative ART-R-associated genes in the ≥ 80% and < 80% PRR groups. Variants were identified by WGS analysis relative to the 3D7 reference sequence in K13 outside the propeller domain, Coronin, KIC4, KIC5, KIC7, PX1, AP2-Mu, and UBP1. Amino acid positions are numbered according to the 3D7 reference sequence. Asterisks indicate substitutions or indels showing a nominally significant difference in frequency between the two PRR groups by Fisher’s exact test (*P* < 0.05). None of these differences remained significant after multiple testing correction

A total of eight genes that were reported to be directly involved in ART-R were examined, including *k13*, *coronin*, *kic4*, *kic5*, *kic7*, *px1*, *ap2-mu*, and *ubp1* (Fig. 3, Table S1, Table S2-1). For the *k13* gene, clear sequences were obtained from all 31 samples and supported the Sanger sequencing results, focusing on the K13 propeller domain, except MAL040 that showed no g1734t (A578S) substitution by WGS. This was presumably attributable to a reduction in the proportion of clones containing the A578S substitution during culture, although the contribution of this substitution remains unclear. Two previously unreported nonsynonymous polymorphisms located upstream of the propeller domain were identified, resulting in amino acid substitutions A359V (3.2% at the clone level; hereafter, frequencies are described at the clone level) and I394M (1.6%). Additionally, several known variants were identified, including F45Y (3.2%), T149S (1.6%), K189T (12.9%), K189N (1.6%), and R255K (4.8%). Although K189T is not an associated ART partial-resistance marker, this substitution is specifically mentioned by WHO as a frequently reported mutation outside the propeller domain. Two clones contained seven Asn residues, and one clone contained eight Asn residues, between H136 and L143 of the 3D7 K13 sequence, which contains only six Asn residues. An increased number of Asn residues at this position has been associated with clinical resistance and in vitro tolerance against dihydroartemisinin (DHA) [27].

In the *coronin* gene, one previously unreported nonsynonymous substitution was identified, resulting D528N (3.2%), and multiple known variants, such as P76S (3.2%), S183G (79.0%), V424I (8.1%), and F434L (6.5%), which are commonly found in the African *P. falciparum* population. In the *kic4* gene, six novel nonsynonymous substitutions, resulting in amino acid substitutions of D16N (3.2%), E285K (3.2%), D535I (1.6%), K561E (3.2%), K578N (3.2%), and T614K (1.6%) were identified. Similarly, in the *kic5* gene, eight unreported nonsynonymous substitutions were detected, resulting in E171G (3.2%), T222S (3.2%), D256Y (1.6%), N472S (1.6%), P577L (1.6%), S739I (3.2%), N1046H (3.2%), and S1721A (100%). We found unreported insertion of one Asp residue after Q1310 to expand a stretch of three consecutive Asp residues to four Asp residues between Q1310 and A1314 in 2 isolates. We also identified Asn-repeat length polymorphisms between D1601 and S1613. Although this region contains 11 Asn residues in the 3D7 strain, repeat lengths of 10, 12, 13, and 14 residues were observed in 11 clones at frequencies of 4.8%, 1.6%, 8.1%, and 1.6%, respectively. For the *kic7* gene, two novel nonsynonymous variants were identified, resulting in the substitutions M319I (3.2%) and M512V (3.2%), along with known variants N434S (8.1%) and S576N (8.1%). In the *px1* gene, in addition to previously reported 21 nonsynonymous variants, several novel variants were identified, including Y5H (3.2%), Y601D (1.6%), D628Y (1.6%), D747K (3.2%), and G1229V (3.2%). We also identified unreported Asp-repeat length polymorphisms between N648 and E653. This region contains 4 Asp residues in the 3D7 strain, whereas 5 clones (8.1%) contained 5 residues and one clone (1.6%) contained 6 residues. Other previously unreported insertions were a KD insertion between D746 and D747 in two clones (3.2%), an N983 deletion in one clone (1.6%), and an NN insertion after N1000 in one clone (1.6%), expanding the Asn-repeat region between D982 and L1001 from 10 to 12 residues. In the *ap2-mu* gene, the S160N variant that is associated with in vitro DHA tolerance was identified in 5 clones (8.1%) [7] with two other reported nonsynonymous variants; K199T (21.0%) and A337S (3.2%). Previously reported insertion of Asn residues was also detected between I226 and N227 in 18 clones (one-Asn and two-Asn insertions accounted for 27.4% and 1.6%, respectively), one Asn insertion between N324 and T325 in five clones (8.1%), and one Asn insertion between S326 and N327 in one clone (1.6%) [6]. The *ubp1* gene showed 78 nonsynonymous polymorphic sites. In addition to the previously reported 61 amino acid variants [28], following 17 unreported amino acid variants were identified; S181N (1.6%), D333E (3.2%), D334N (1.6%), N394S (1.6%), D465H (3.2%), K785N (3.2%), S1328R (3.2%), L1401F (1.6%), R1463H (1.6%), S1472Y (3.2%), N1966K (3.2%), W2031C (3.2%), N2569Y (3.2%), F2572Y (3.2%), V2611L (1.6%), K2679Q (1.6%), and N3035S (1.6%). Among the previously reported variants, E1528D was detected in five clones (8.1%). Although not recognized as a WHO-validated molecular marker of artemisinin resistance, this substitution was reported to be significantly enriched after artemisinin combination therapy (ACT) in Kenya [6]. D1525E, a substitution observed in Africa with an unclear association with artemisinin resistance, was also detected in two clones (3.2%) in the present study. UBP1 indels were detected 16 positions. The most frequent changes were an insertion of DDNN between D333 and D334 in 15 clones (24.2%), deletion of N474 or N474–N475 in 15 clones (24.2%), deletion of N199/N200 in 14 clones (22.6%), insertion of DD or G between K785 and D786 in 13 clones (21.0%). Other relatively common indels included insertions of N/NN/NNN between H17 and N18 in nine clones (14.5%), SINN between D226 and S227 in nine clones (14.5%), K/N/NN between K1705 and N1706 in nine clones (14.5%), deletion of N18/N19 in eight clones (14.5%), and S between M1 and S2 (12.9%). The remaining indels were detected in seven or fewer clones. Insertion of aatcatgtg between between a9270 and a9271 resulted premature termination at K2817 (22.6%) (Fig. 3, Table S1, Table S2-1).

### Substitutions of three genes indirectly associated with ART-R

**Fig. 4:**
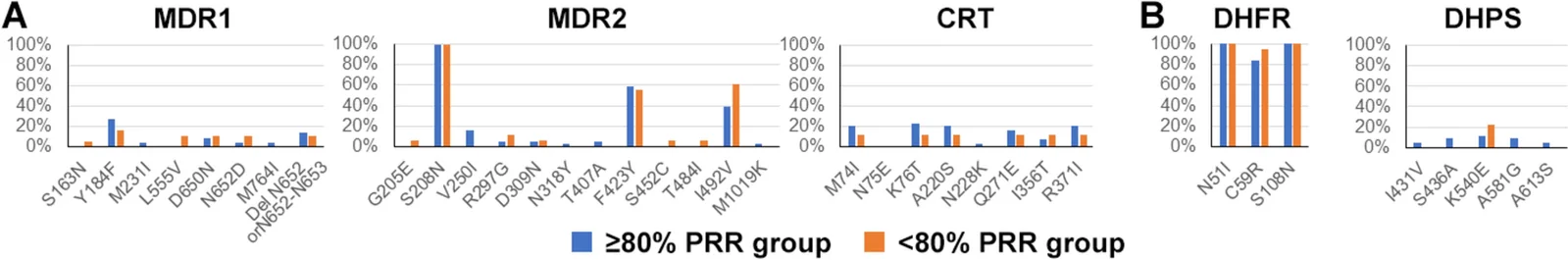
Frequencies of parasite clones carrying amino acid substitutions or indels in other five genes in the ≥ 80% and < 80% PRR groups. Variants were identified by WGS analysis relative to the 3D7 reference sequence in MDR1, MDR2, CRT, DHFR, and DHPS. Amino acid positions are numbered according to the 3D7 reference sequence. No substitutions or indels were significantly different between the two PRR groups by Fisher’s exact test at *P* < 0.05

Then, three genes reported to be indirectly involved in ART-R were examined; *mdr1*, *mdr2*, and *crt* (Fig. 4A, Table S1, Table S2-2). Four *mdr1* variants resulting in amino acid changes, S163N (1.6%), M231I (3.2%), L555V (3.2%), and M764I (3.2%), were newly identified in this study. The S163N variant was identified for the first time in *P. falciparum*, but the amino acid at the equivalent position has been reported as N in the *P. vivax* ortholog [29]. Reported variants such as Y184F (24.2%), D650N (9.7%), and N652D (6.5%) were also identified. The Y184F substitution was reported to be associated with reduced susceptibility to lumefantrine (LUM), particularly as part of the *Pfmdr1* N86/184F/D1246 haplotype [30]. N652 or N652-N653 deletions were detected in 8 clones (12.9%). N86Y and D1246Y variants that modulate susceptibility to CQ and/or AQ were not detected, and all isolates showed 3D7-type N86 and D1246 in this study, which have been reported to have reduced sensitivity to LUM [31]. On the *mdr2* gene, five novel nonsynonymous polymorphisms were seen, resulting in amino changes G205E (1.6%), R297G (6.5%), N318Y (1.6%), T407A (3.2%), and S452C (1.6%), alongside seven known variants S208N (100%), V250I (11.3%), D309N (4.8%), F423Y (58.1%), T484I (1.6%), I492V (45.2%), and M1019K (1.6%). The T484I substituion has been reported as a genetic background marker of artemisinin-resistant founder populations in Southeast Asia, although its direct contribution to artemisinin resistance has not been functionally supported [32]. *Pfmdr2* F423Y was independently associated with in vitro pyrimethamine (PYR) resistance, regardless of PYR-resistance mutations in *dhfr* [33]. In the *crt* gene, one novel variant, N228K (1.6%), and eight known variants were detected, resulting in amino changes M74I (17.7%), N75E (17.7%), K76T (19.4%), A220S (17.7%), Q271E (14.5%), I356T (8.1%), and R371I (17.7%). Codons 74, 75, and 76 in the *crt* gene are well documented to be associated with CQ resistance in Africa and to define the sensitive-type haplotype MNK (M74, N75, K76) and the triple-resistant mutant IET (74I, 75E, 76T) [34]. In this study, the frequencies of major and minor nucleotides at *crt* codons 74, 75, and 76 were clearly distinguishable in each isolate; therefore, amino acid haplotype frequencies across these three codons were estimated accordingly. Overall, 80.6% of the parasites carried the CQ-sensitive MNK haplotype, whereas the CQ-resistant IET haplotype accounted for 17.7% (Table 4). In addition, one clone carried the MNT haplotype.

**Table 4:** Amino acid haplotype frequencies of CRT, DHFR, and DHPS and combination of the DHFR and DHPS

Protein	Haplotype		Frequency
CRT	M74 + N75 + K76 (sensitive 3D7 type)		80.6% (50/62)
	(M74I + N75E + K76T) + A220S + Q271E + I356T + R371I		8.1% (5/62)
	(M74I + N75E + K76T) + A220S + Q271E + R371I		6.5% (4/62)
	(M74I + N75E + K76T) + A220S + R371I		3.2% (2/62)
DHFR	N51I + C59R + S108N (triple mutant)		87.1% (54/62)
	N51I + S108N (double mutant)		12.9% (8/62)
DHPS	I431 + S436 + A437 + K540 + A581 + A613 (sensitive 3D7 type)		79.0% (49/62)
	K540E		11.3% (7/62)
	S436A		3.2% (2/62)
	K540E + A581G		3.2% (2/62)
	I431V + S436A + A581G + A613S		3.2% (2/62)
DHFR + DHPS	*DHFR*	*DHPS*	
	N51I + C59R + S108N	I431 + S436 + A437 + K540 + A581 + A613	69.4–71.0% (43/62–44/62)
	N51I + C59R + S108N	K540E	12.9–14.5% (8/62–9/62)
	N51I + C59R + S108N	K540E + A581G	3.2% (2/62)
	N51I + C59R + S108N	I431V + S436A + A581G + A613S	3.2% (2/62)
	N51I + S108N	I431 + S436 + A437 + K540 + A581 + A613	9.7% (6/62)
	N51I + S108N	S436A	3.2% (2/62)
	N51I + S108N	K540E	0–1.6% (0/62–1/62)

*crt*, chloroquine resistance transporter (PF3D7_0709000); *dhfr* is a part of *dhfr-ts*, dihydrofolate reductase-thymidylate synthase (PF3D7_0417200); *dhps* is a part of *pppk-dhps*, hydroxymethyldihydropterin pyrophosphokinase-dihydropteroate synthase (PF3D7_0810800). Amino acid positions are based on the 3D7 reference sequence

### Substitutions of genes associated with resistance against PYR and sulfadoxine

We analyzed mutations in the gene regions encoding DHFR domain of DHFR-TS (*dhfr*) and the DHPS domain of PPPK-DHPS (*dhps*), both of which are associated with SP resistance (Fig. 4B,, Table S1, Table S2, 3) [35]. In the *dhfr* gene region, PYR-resistance-associate variants N51I and S108N substitutions were detected in all clones, whereas C59R was detected at a frequency of 87.1%. Accordingly, the high-resistance triple haplotype N51I/C59R/S108N was observed at 87.1%. The DHFR I164L variant, which confers high-level resistance to antifolate drugs, was not observed in this study. In the *dhps* gene region, five nonsynonymous variants associated with sulfadoxine resistance were identified at low frequencies: I431V (3.2%), S436A (6.4%), K540E (16.1%), A581G (6.4%), and A613S (3.2%) [36]. The DHPS A437G, one of the key variants mediating sulfadoxine resistance, was not found in this study.

We subsequently analyzed the frequencies of combined DHFR–DHPS haplotypes. For all isolates except K026, DHFR and DHPS haplotypes could be combined without ambiguity. In isolate K026, the read frequencies of DHPS K540K and K540E were nearly equal, at 45% and 54%, respectively; therefore, their linkage with DHFR C59C or C59R could not be determined. Haplotype frequencies were therefore calculated by taking both possible combinations into account. Combined DHFR–DHPS haplotype analysis showed that the most clones (69.4–71.0%, depending on the possible haplotype assignment for isolate K026) carried the DHFR triple haplotype N51I/C59R/S108N together with a DHPS sensitive-type haplotype. The quadruple resistant haplotype, consisting of the DHFR triple haplotype plus DHPS K540E, was observed in 12.9–14.5% of clones. Haplotypes carrying additional DHPS substitutions were uncommon: two clones carried the DHFR triple haplotype combined with DHPS K540E/A581G substitutions, and two clones carried the DHFR triple haplotype combined with DHPS I431V/S436A/A581G/A613S (Table 4).

### Nucleotide polymorphisms with differential allele frequencies between the ≥ 80% and < 80% PRR groups

Among 259 polymorphic sites and 27 indels in 11 genes that are directly or indirectly associated with ART-R, four nonsynonymous and three synonymous sites showed nominally significant differences in amino acid or nucleotide frequencies between the ≥ 80% and < 80% PRR groups by Fisher’s exact test. K13 K189T and UBP1 K1950E were more frequent in the < 80% PRR group (*P* = 0.039 and *P* = 0.022, respectively) and Coronin S183G and UBP1 R2238K were less frequent in the < 80% PRR group (*P* = 0.04 and *P* = 0.005, respectively). *kic4* g2589a and *ap2-mu* a300c were more frequent in the < 80% PRR group (*P* = 0.01 and *P* = 0.00007, respectively) and *kic5* c1890t was less frequent in the < 80% group (*P* = 0.049) (Fig. 3). However, only *ap2-mu* a300c remained significant after correction for multiple testing across 291 sites (adjusted *P* = 0.0189). Other sites should be interpreted as candidate polymorphisms requiring further validation (Table 5, Table S2).

**Table 5:** Nucleotide polymorphisms with differential allele frequencies between the ≥ 80% and < 80% PRR groups

	Nucleotide change	Amino acid change	≥ 80% PRR group	< 80% PRR group	Two-tailed Fisher's exact test	After FDR correction
*k13*	a566c	K189T	6.8% (3/44)	27.8% (5/18)	*P* = 0.0392	not significant
*coronin*	a547g	S183G	86.4% (38/44)	61.1% (11/18)	*P* = 0.0399	not significant
*ubp1*	a5848g	K1950E	0.0% (0/44)	16.7% (3/18)	*P* = 0.0216	not significant
*ubp1*	g6713a	R2238K	100.0% (44/44)	77.8% (14/18)	*P* = 0.0055	not significant
*kic4*	g2589a	(–)	9.1% (4/44)	38.9%(7/18)	*P* = 0.0097	not significant
*kic5*	c1890t	(–)	20.5% (9/44)	0.0% (0/18)	*P* = 0.0486	not significant
*ap2-mu*	a300c	(–)	0.0% (0/44)	38.9% (7/18)	*P* = 0.000065	*P* = 0.0189

A plausible interpretation is that *ap2-mu* a300c may represent a lineage marker linked to other functional variants. Among the four isolates carrying the *ap2-mu* a300c mutation, two also carried an a479g mutation located 179 bp downstream of a300c, and two carried an a489g mutation located 189 bp downstream. We examined whether linkage disequilibrium was present between nucleotide position 300 and these sites; however, the corresponding r^2^ values were low (0.0722 and 0.0069, respectively), making it difficult to argue that linkage disequilibrium is present for sites located approximately 200 bp from a300c. The coding region extends 300 bp upstream of a300c, but no polymorphic sites were found in this region. Furthermore, we examined 200 bp of the 5′ UTR, but found no characteristic variants shared by the four isolates carrying the *ap2-mu* a300c mutation (Fig. S2). Taken together, it seems unlikely that another true causal mutation exists nearby and that a300c showed a significant difference merely as a result of a hitchhiking effect.

## Discussion

In this study, we characterized polymorphisms in antimalarial drug resistance-associated genes in *P. falciparum* clinical isolates from Kinshasa, DRC, with particular attention to markers potentially associated with artemisinin resistance. Among 303 enrolled patients who were positive by malaria rapid diagnostic test (RDT), parasites were confirmed by microsocpy in 189 samples. This discrepancy is likely explained by a combination of submicroscopic parasitemia, persistence of circulating parasite antigen after recent infection, and variation in sample quality or microscopic sensitivity. Among microscopy-confirmed *P. falciparum* infections, 11 patients showed < 80% reduction in parasitemia one day after AL treatment. These samples were considered to represent delayed early parasite clearance, but not confirmed clinical artemisinin resistance.

In the WHO Compendium of molecular markers for antimalarial drug resistance, published on 10 December 2025, 14 K13 mutations are classified as validated markers of ART partial resistance, and six additional K13 mutations are classified as candidate markers associated with delayed parasite clearance [37]. However, no validated or candidate artemisinin resistance-associated K13 propeller mutations were detected among 171 successfully sequenced samples, including those from patients with < 80% day 1 parasitaemia reduction. This finding suggests that canonical K13 propeller-mediated artemisinin partial resistance was not evident in the study population at the time of sampling. One isolate carried the K13 A578S substitution, but this variant has been reported in multiple Africa and Asia parasite population and has not been shown to confer artemisinin resistance in gene-edited transgenic parasites [24, 25]. Therefore, the present data do not support the circulation of established K13-mediated artemisinin-resistant parasites in Kinshasa.

However, absence of validated K13 propeller mutations does not exclude the possibility of altered artemisinin response through other mechanisms. Clinical or parasitological delayed clearance in Africa may occur in the absence of known K13 resistance mutations, and parasite genetic backgrounds outside the K13 propeller domain may influence artemisinin susceptibility. In this study, two previously unreported K13 nonsynonymous substitutions, A359V and I394M, were detected upstream of the propeller domain. Although their biological significance is unknown, their location outside the classical propeller domain indicates that they should not be interpreted as established artemisinin resistance markers. They are better regarded as variants of interest requiring phenotypic and functional validation.

We also detected variation in the number of Asn residues between H136 and L143 of K13. The 3D7 reference sequence contains six Asn residues in this region, whereas three clones in this study carried seven or eight residues. Notably, a previous study reported that 86% of *P. falciparum* isolates collected from the China-Myanmar border between 2008 and 2013 possessed eight Asn residues at this site, and an increased number of Asn residues was significantly associated with day 3 parasite positivity after treatment with artemisinin-family drugs and with increased ring-stage survival following DHA exposure [27]. Subsequently, Xiang et al. generated parasite lines in which the 3D7 strain was genetically modified to contain seven, eight, or nine Asn residues at this position and evaluated them by RSA. They found that increasing the number of Asn residues to seven or eight did not result in an obvious increase in DHA-associated ring-stage survival, whereas the nine-Asn line showed a significantly increased survival rate, albeit with an accompanying fitness cost [38]. These findings suggest that variation of seven or eight Asn residues alone appears to be limited under in vitro conditions. From this perspective, the detection of seven- or eight- Asn variants in three DRC clones is noteworthy, even though these clones belonged to the ≥ 80% PRR group. Continued monitoring of Asn-repeat lengtha this site may therefore be warranted. In addition, more than 10% of *P. falciparum* isolates from Dakar, Senegal, have been reported to carry seven Asn residues at this position [39], indicating that such repeat-length variation is also present in African parasite population.

WGS of 31 culture-adapted isolates revealed extensive diversity in non-*k13* genes implicated in artemisinin response, including *coronin*, *kic4*, *kic5*, *kic7*, *px1*, *ap2-mu*, and *ubp1*. Several of these findings are noteworthy. In PX1, PIN haplotype (L1222P, M1701I, and D1705N) has recently been shown to reduced ex vivo susceptibility to DHA, LUM, and MQ in Uganda [10]. In the present study, the M1701I and D1705N substitutions were detected as part of LIN (L1222, 1701I, and 1705N) or LID (L1222, 1701I, and D1705) haplotypes. However, the full PIN haplotype was not observed in this study. Niare et al. (2025) reported that among 364 DRC *P. falciparum* sequences in the Pf6 WGS dataset, only 3 carried the PIN haplotype, two in 2012 and one in 2014, while the LIN haplotype was 9.3% with LID haplotype of approximately 24% [10, 40]. These are consistent with our findings; the LIN haplotype in four clones (6.5%) and the LID haplotype in 11 clones (17.7%). The AP2-Mu S160N, previously implicated in altered in vitro parasite response to DHA, was also detected at low frequency (8.1%) [7]. In UBP1, E1528D was observed at low frequency (8.1%); this variant has been discussed as candidate markers associated with ACT selection [6]. These variants are not WHO-validated artemisinin resistance markers, but their presence supports the need to expand surveillance beyond the K13 propeller domain.

**Fig. 5:**
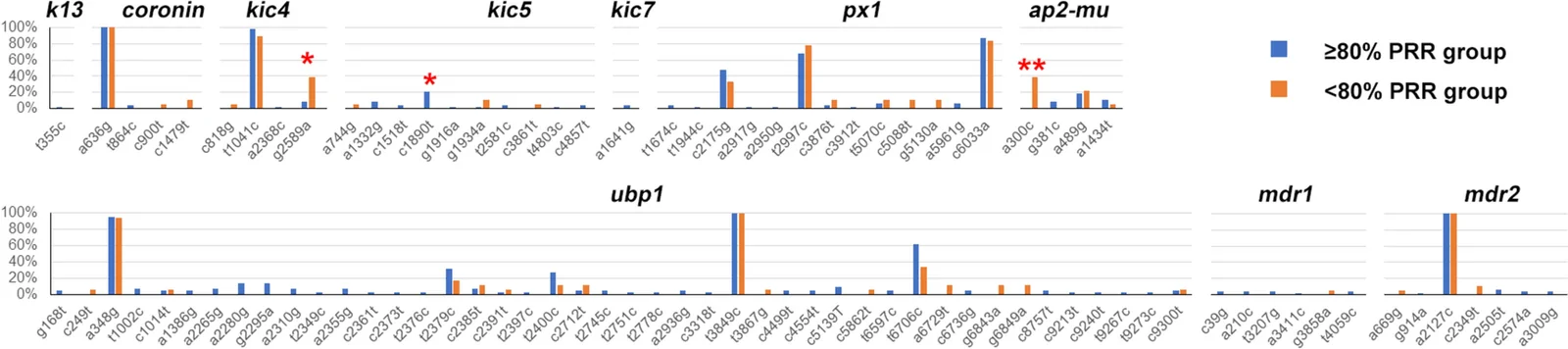
Frequencies of parasite clones carrying synonymous substitutions in the ≥ 80% and < 80% PRR groups. Variants were identified by WGS analysis relative to the 3D7 reference sequence in *k13*, *coronin*, *kic4*, *kic5*, *kic7*, *px1*, *ap2-mu*, *ubp1*, *mdr1*, and *mdr2*. No synonymous substitutions were detected in *crt*, *dhfr* or *dhps*. Nucleotide positions are numbered according to the 3D7 reference sequence. Asterisks indicate substitutions with a nominally significant difference in frequency between the two PRR groups by Fisher’s exact test (*P* < 0.05). Double asterisks indicate the substitution that remained significant after multiple testing correction (adjusted *P* = 0.0189)

When allele frequencies were compared between the ≥ 80% and < 80% PRR groups, several polymorphisms showed nominally significant differences. After correction for multiple testing, only the synonymous *ap2-mu* a300c polymorphism remained significant (Fig. 5, Table S1, Table S2–4). Because this is a synonymous substitution, it is unlikely to directly alter protein function. Because no clear linkage disequilibrium was observed between a300c and nearby polymorphic sites, the hypothesis that a300c was selected through a hitchhiking effect is not supported. Another possibility is that this synonymous codon change may affect mRNA stability, translation efficiency, co-translational folding, and potentially RNA modification patterns. In *P. falciparum*, whose genome is strongly biased toward AT-rich codons, the ata-to-atc change due to the a300c substitution represents a shift from a frequently used Ile codon to a relatively rare one, with codon usage frequencies of 50.0% and 6.3%, respectively, based on 4023 open reading frames of the 3D7 strain [41]. Nevertheless, its functional consequence would require experimental validation. Finally, the observed association may also reflect sampling variation in a limited dataset. Therefore, this result should be interpreted as a candidate molecular signal rather than evidence of a causal artemisinin resistance marker. Larger studies combining clinical clearance data, parasite genotyping, and ex vivo drug susceptibility assays are required to determine whether this association is reproducible and biologically meaningful.

Variants in genes associated with partner-drug response were also detected. In MDR1, Y184F was present, whereas N86Y and D1246Y were not observed. The N86/184F/D1246 haplotype has been associated with altered LUM susceptibility or selection after AL treatment in several African settings [30]. Although this study did not directly assess LUM susceptibility, the observed MDR1 profile suggests that partner-drug selection should continue to be monitored in Kinshasa. No copy number increase was detected in the analyzed resistance-associated genes, suggesting that copy number-mediated resistance involving these loci was not evident in this sample set.

Although the CQ-sensitive CRT MNK haplotype was predominant, the CQ resistance-associated IET haplotype remained detectable. This suggests the continued persistence of CQ-resistant parasites in Kinshasa at a moderate frequency, despite the withdrawal of CQ as first-line treatment. The CRT K76T prevalence observed in this study in 2022 was 19.4%, broadly comparable to the 27.3% reported among Kinshasa samples collected in 2019 by Nundu et al. [42].

These findings suggest that the efficacy of SP for intermittent preventive treatment in pregnancy may be largely driven by sulfadoxine. Therefore, there is an urgent need to develop and implement safe, effective alternative antimalarial drugs for use in pregnant women, to replace SP, or at least PYR.

The antifolate resistance profile showed a high prevalence of the DHFR triple-mutant haplotype N51I/C59R/S108N (87.1%), whereas DHPS resistance-associated substitutions were detected at lower frequencies. The DHFR I164L mutation was not detected, and the key DHPS A437G mutation was also absent. The most common combined DHFR*–*DHPS profile was the DHFR triple mutant with a DHPS sensitive-type haplotype, followed by the DHFR triple mutant plus DHPS K540E. These findings indicate that PYR resistance-associated mutations are highly prevalent in this population, while the accumulation of multiple DHPS mutations remains less common. A broadly similar pattern was reported in Kinshasa in 2019, where the DHFR IRN haplotype and DHPS K540E were detected in 94% and 19.0% of samples, respectively [42]. This suggests that the allele frequency distribution of these antifolate resistance-associated genes remained broadly stable in Kinshasa over the 3–4-year interval between the two studies, despite differences in the study populations: the earlier study focused on school-aged children, whereas the present study included a broader patient population [42, 43]. Together, these findings suggest that the remaining efficacy of SP for intermittent preventive treatment in pregnancy may be primarily sustained by the sulfadoxine component. Continued surveillance of DHPS mutations, especially K540E, A581G, A613S, and of the emergence of higher-order resistant haplotypes is therefore warranted.

The successfully established culture-adapted *P. falciparum* field isolates represent a valuable resource for future integrated phenotypic and genotypic investigations. However, it should be acknowledged that, if parasites carrying resistance-associated alleles have a higher fitness cost than those carrying wild-type alleles in the absence of drug pressure, the proportion of resistant parasites may decline during culture in mixed infections of drug-sensitive and drug-resistant clones. Because culture and WGS were performed without cloning in this study, the detection of resistance-associated alleles may therefore have been underestimated. It should also be noted that, because the sample size of 31 is too small to be regarded as representative of the parasite population circulating in Kinshasa, conclusions drawn from the WGS data should be interpreted as exploratory and preliminary.

This study has some limitations. First, the WGS analysis was based on only 31 culture-adapted isolates, including nine from the < 80% PRR group, and therefore had limited power to identify robust genotype–phenotype associations. Second, culture adaptation may alter clone composition in mixed infections. This is particularly relevant because the K13 A578S variant detected by DBS-based Sanger sequencing was not detected by WGS after culture, suggesting that minor clones may be lost or reduced during culture adaptation. Third, WGS was performed without parasite cloning. The assumption that each isolate comprised two clones was a practical approach for allele frequency estimation, but it cannot fully resolve complex mixed infections or reconstruct haplotypes with certainty. Fourth, day 1 PRR is an exploratory indicator of early parasite clearance and is not equivalent to WHO-defined delayed parasite clearance or confirmed clinical artemisinin resistance.

Despite these limitations, this study provides useful information on the current genetic landscape of *P. falciparum* drug resistance-associated genes in Kinshasa. The absence of validated *k13* propeller markers is reassuring, but the detection of diverse non-*k13* candidate variants, including variants in *px1*, *ap2-mu*, and *ubp1*, indicates that molecular surveillance should not be restricted to the K13 propeller domain. Integration of expanded molecular marker panels with therapeutic efficacy studies, parasite clearance analysis, and ex vivo drug susceptibility testing will be essential for early detection of emerging artemisinin and partner-drug resistance in the DRC.

## Conclusions

No validated or candidate K13 propeller mutations associated with ART resistance were detected among *P. falciparum* isolates from Kinshasa, including samples from patients with < 80% parasitaemia reduction one day after AL treatment. One isolate carried K13 A578S, a variant previously shown not to confer artemisinin resistance. WGS revealed diverse known and novel polymorphisms in drug resistance-associated genes, including non-*k13* loci implicated in ART response. Variants in *px1*, *ap2-mu*, and *ubp1* may represent candidate signals for altered ACT response, but their biological relevance remains unproven. The synonymous *ap2-mu* a300c polymorphism was associated with the < 80% PRR group after multiple-testing correction, but this finding requires validation in larger studies with phenotypic drug susceptibility data. High frequencies of DHFR antifolate resistance mutations, moderate persistence of CRT CQ resistance-associated haplotypes, and detectable DHPS mutations indicate continued selection pressure on multiple antimalarial resistance pathways. These findings support continued molecular surveillance in Kinshasa using both *k13* and non-*k13* marker panels, combined with clinical and ex vivo phenotypic monitoring.

## Supplementary Information

Additional file 1. Figure S1. Average read depth of culture-adapted strains' sequences. Samples were divided into 2 groups based on the status of day 1 parasitemia reduction rate. **Figure S2**. Alignment of the 200-bp region upstream of the *ap2-mu* gene locus. Description: Samples were stratified into the ≥ 80% and < 80% parasitemia reduction rate (PRR) groups, and isolates carrying the a300c mutation are indicated in red.Summary of the frequencies of detected nonsynonymous substitutions and indels in the samples.

Additional file 2. Table S1. Summary of the nucleotide substitutions with the read number and the ratio. All substitutions and indels detected in 13 genes examined for 31 Kinshasa isolates are summarized with the read number and the ratio.

Additional file 3.

## Data Availability

Whole genome sequence data reported are available in the DDBJ Sequenced Read Archive under the accession numbers DRR894079—DRR894109. The data have been deposited with links to BioProject accession number PRJDB39668 in the DDBJ BioProject database, and BioSample metadata are available in the DDBJ BioSample database under accession number SAMD01782957—SAMD01782987. Sanger sequence data have been deposited with links to BioProject accession number PRJDB39668 in the DDBJ BioProject database, and BioSample metadata are available in the DDBJ BioSample database under accession number SAMD01791115—SAMD01791285.

## Abbreviations

AA: Amino acid
ACT: Artemisinin combination therapy
AL: Artemether-lumefantrine
ART-R: Artemisinin resistance
CRT: Chloroquine resistance transporter
DHA: Dihydroartemisinin
DHFR-TS: Dihydrofolate reductase–thymidylate synthase
DNA: Deoxyribonucleic acid
DRC: Democratic Republic of the Congo
KIC: Kelch13 interaction candidates
K13: Kelch 13 protein
MDR1: Multidrug resistance 1
MDR2: Multidrug resistance 2
PCR: Polymerase chain reaction
PPPK-DHPS: Hydroxymethyldihydropterin pyrophosphokinase–dihydropteroate synthase
RDT: Rapid diagnostic test
PRR: Parasitemia reduction rate
PYR: Pyrimethamine
SP: Sulfadoxine-pyrimethamine
UBP1: Ubiquitin binding protein 1
WGS: Whole genome sequence
WHO: World Health Organization
