# Supplementary material for: Drug resistance gene polymorphisms of Plasmodium falciparum clinical isolates from Kinshasa, Democratic Republic of the Congo
Source: Trop Med Health. 2026 Jul 7;54:158. doi: 10.1186/s41182-026-01022-5 (PMC13491791; doi:10.1186/s41182-026-01022-5)

Figure S1. **Average read depth of culture-adapted strains' sequences**. Samples were divided into 2 groups based on the status of day 1 parasitemia reduction rate.

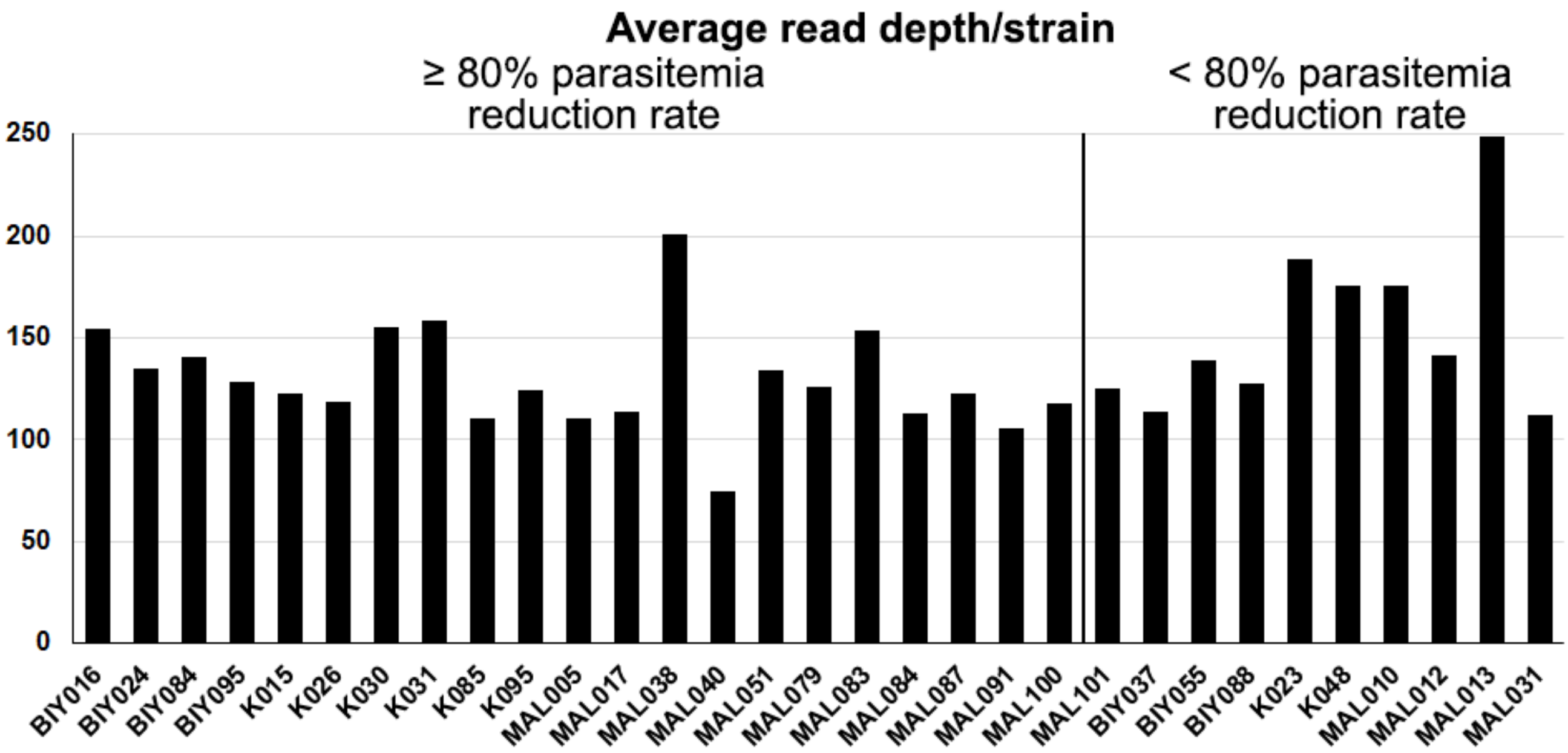

Figure S2. **Alignment of the 200-bp region upstream of the *ap2-mu* gene locus**. Samples were stratified into the ≥ 80% and < 80% parasitemia reduction rate (PRR) groups, and isolates carrying the a300c mutation are indicated in red.Summary of the frequencies of detected nonsynonymous substitutions and indels in the samples.

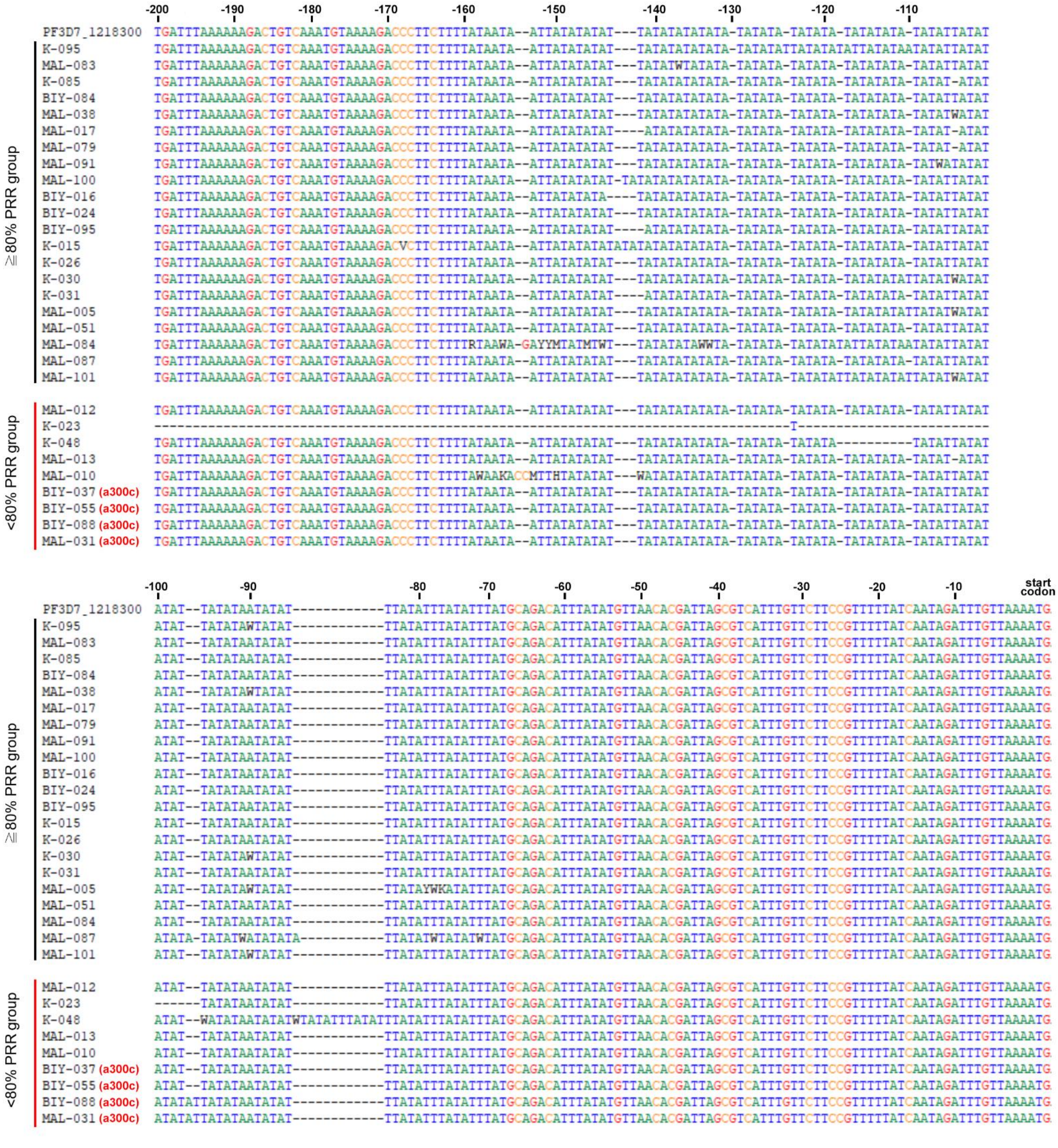

Supplement: Supplementary file 1 [file 41182_2026_1022_MOESM1_ESM.pdf]
